# Supplementary material for: Comparative Transcriptomic Analyses for the Optimization of Thawing Regimes during Conventional Cryopreservation of Mature and Immature Human Testicular Tissue
Source: Int J Mol Sci. 2023 Dec 22;25(1):214. doi: 10.3390/ijms25010214 (PMC10778995; doi:10.3390/ijms25010214)
Supplement: Supplementary file 1 [file ijms-25-00214-s001.zip › ijms-2737474-supplementary.pdf]

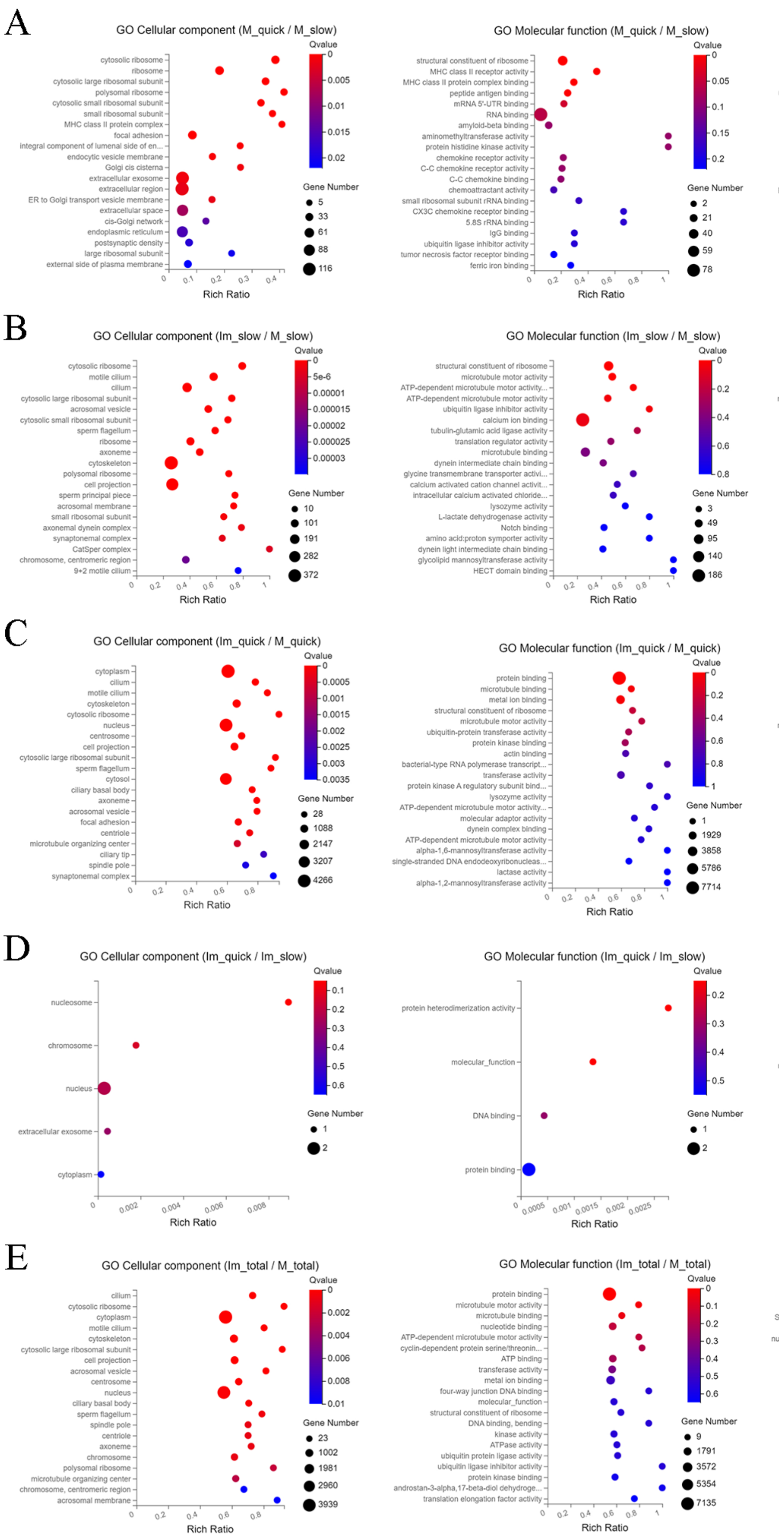

Figure S1. GO Cellular component and Molecular function enrichment bubble chart for different groups.
